# Paclitaxel-Based Chemotherapy Targeting Cancer Stem Cells from Mono- to Combination Therapy

**DOI:** 10.3390/biomedicines9050500

**Published:** 2021-05-02

**Authors:** Hend M. Nawara, Said M. Afify, Ghmkin Hassan, Maram H. Zahra, Akimasa Seno, Masaharu Seno

**Affiliations:** 1 Department of Biotechnology and Drug Discovery, Graduate School of Interdisciplinary Science and Engineering in Health Systems, Okayama University, Okayama 700-8530, Japan; ps3q5xgk@s.okayama-u.ac.jp (H.M.N.); saidafify@s.okayama-u.ac.jp (S.M.A.); pthz2c4o@s.okayama-u.ac.jp (G.H.); maram@okayama-u.ac.jp (M.H.Z.); aseno@okayama-u.ac.jp (A.S.); 2 Division of Biochemistry, Chemistry Department, Faculty of Science, Menoufia University, Menoufia 32511, Egypt; 3 Department of Microbiology and Biochemistry, Faculty of Pharmacy, Damascus University, Damascus 10769, Syria

**Keywords:** paclitaxel, microtubule targeting agent, anticancer, cancer stem cells, combination therapy

## Abstract

Paclitaxel (PTX) is a chemotherapeutical agent commonly used to treat several kinds of cancer. PTX is known as a microtubule-targeting agent with a primary molecular mechanism that disrupts the dynamics of microtubules and induces mitotic arrest and cell death. Simultaneously, other mechanisms have been evaluated in many studies. Since the anticancer activity of PTX was discovered, it has been used to treat many cancer patients and has become one of the most extensively used anticancer drugs. Regrettably, the resistance of cancer to PTX is considered an extensive obstacle in clinical applications and is one of the major causes of death correlated with treatment failure. Therefore, the combination of PTX with other drugs could lead to efficient therapeutic strategies. Here, we summarize the mechanisms of PTX, and the current studies focusing on PTX and review promising combinations.

## 1. Introduction

Cancer is usually characterized by unlimited growth and insensitivity to the growth-preventing signals from other tissues, resulting in invasion into surrounding tissues and metastasis to distant organs and/or tissues [1,2]. Cancer tissues contain several distinct cellular subpopulations reflecting the heterogeneity arising from a rare population of cells, which are hypothesized to be cancer stem cells (CSCs) with the abilities of tumorigenesis, self-renewal, and differentiation [3,4,5]. The ratio of CSCs to non-CSCs in a tumor correlates with poor clinical prognosis [6]. CSCs are chemo-resistant, not only surviving after chemotherapy [7,8,9,10,11,12,13] but also causing recurrence and metastasis [14,15], while chemotherapy usually eradicates the bulk population of non-CSC tumor cells. In this context, promising novel strategies to treat cancers inhibiting the ability of CSC to self-renew, invade, and metastasize are urgently needed [16,17].

PTX is found in the bark extract of the Pacific Yew tree, *Taxus brevifolia*. The isolation and identification of PTX were achieved by Wall and Wani [18]. PTX exhibited not only strong cytotoxic activities against the growth of human cervical cancer cell line HeLa cells at nanomolar concentrations but also cell-cycle arrest in the mitotic (M) phase without disturbing the synthesis (S) phase [19,20]. PTX has been demonstrated as an effective anticancer agent against lung, breast, ovarian, leukopenia, and liver cancer in various studies [21,22,23,24,25,26]. Thus, PTX was approved by the FDA in 1992 [27].

In addition to targeting tubulin-inducing cell-cycle arrest, PTX appears to play a role in enhancing signaling factors in the treatment of cancer [28,29,30,31,32,33,34,35,36]. For example, PTX is also known to induce the production of IL-12 p40, which is the homodimer of the soluble receptor in the macrophages of tumor-bearing hosts, significantly down-regulating tumor growth [37]. PTX also reduces glycolysis in melanoma cells [38]. PTX seems feasible for the treatment of cancer even if the target cells are resistant to chemotherapy.

Several chemotherapeutic attempts have been demonstrated to evaluate the combination of conventional drugs as a CSC-targeting strategy [6,39]. However, the heterogeneity of cancer tissue, high plasticity of CSCs, and complexity of the targeting molecules due to the diversity of cancers have only amplified the challenges in identifying suitable drugs [40]. PTX has again been approved by the FDA to be used in combination with other anticancer drugs to treat a wider range of different types of cancers [41]. In this review, we describe the availability of PTX in mono- and combinatorial use in the treatment of different types of cancers.

## 2. Definition of CSCs

CSCs are cancer cells with stem properties of self-renewal, differentiation potential, and malignancy [42,43,44,45,46,47]. CSCs were first recognized and categorized in the bone marrow of AML patients in 1997. They contribute to the heterogeneity in cancer tissue via intra- and inter-tumoral communications between the cells in the microenvironment. Many reports from clinical and laboratory studies support the role of CSCs in drug resistance and cancer metastasis. Poor therapeutic prognosis has been experienced in patients with pancreatic, prostate, liver, breast, and brain tumors linked to CSCs [6,48,49,50].

CSCs generate the original lineage of the cancer cells in addition to the normal stem cells which generate their progeny. Interestingly, some evidence indicates that CSCs occasionally exhibit the ability to transdifferentiate into vascular endothelial cells [51] and other cancer-associated phenotypes [52,53,54]. This demonstrates their ability to transdifferentiate into other lineages of cells endorsing tumor growth and metastasis, not only those providing stromal tissues. This variety of this transdifferentiation is responsible for the hierarchy of the cells, establishing the microenvironment maintaining the cancer tissue. Although many aspects still remain elusive, the transdifferentiation of CSCs into tumor-related cells in the stroma delivers a new aspect of tumor heterogeneity [55]. Stemness properties in proliferation and differentiation are highly dysregulated through different cytoplasmic signaling pathways in CSCs due to genetic and epigenetic changes [47].

## 3. Mechanisms of Resistance in CSCs

CSCs exhibit therapeutic resistance during chemotherapy, recolonizing residual tumors [14]. Ionizing radiation induces the upregulation of CSC markers such as CD133 in glioblastoma xenografts [56] and breast cancer [57], enriching the CSC population. The evidence of CSC enrichment has also been demonstrated by its acquired resistance to one specific drug resulting in cross-resistance to another [58,59,60]. Generally, CSCs evade chemotherapy by expressing multidrug resistance (MDR) transporters via enhanced DNA repair capability, resulting in more effective protection compared to the rest of the tumor cells [61]. The resistance of CSCs to therapy is mediated by strong response and repair pathways against DNA damage. Another special protection mechanism incorporated in them is the regulation and elongation of the G1 phase, allowing CSCs sufficient time for the repair of DNA damage. Moreover, DNA is much more secured in CSCs by the strong scavenging efficiency of reactive oxygen species (ROS).

The factors and/or pathways essential for CSCs to survive and keep growing can enhance the cellular insensitivity to normal chemotherapy. Numerous clinical trials designed to eliminate CSCs are currently ongoing [45]. Nevertheless, CSCs are not of a single-cell phenotype, but rather they are composed of heterogeneous phenotypes, making it enormously hard to predict whether a specific CSC-targeting therapy would be efficient for an individual patient [39,62]. In order to facilitate the development of tailored therapies, it is necessary to identify CSC-specific markers or the regulatory pathways of CSCs in some specific combinations in different phenotypes of cancer cells [63].

The therapeutic resistance of CSCs involves different regulatory mechanisms which make drug combinations more effective by targeting different components and signal pathways, such as drug-efflux pumps, the microenvironment, the quiescent state, and the induction of apoptosis [64].

## 4. Targeting Strategy against CSCs

Although chemotherapy usually eradicates the majority of non-CSC tumor cells, CSCs are not only chemo-resistant but also enriched after chemotherapy [7,8,9,10,11,12,13]. This is because chemotherapy usually targets proliferating cells, while CSCs are often dormant, evading therapy and leading to cancer relapse and metastasis [14,15]. Collectively, CSCs are able to play essential roles in cancer initiation, development, and recurrence [47].

**Figure 1:**
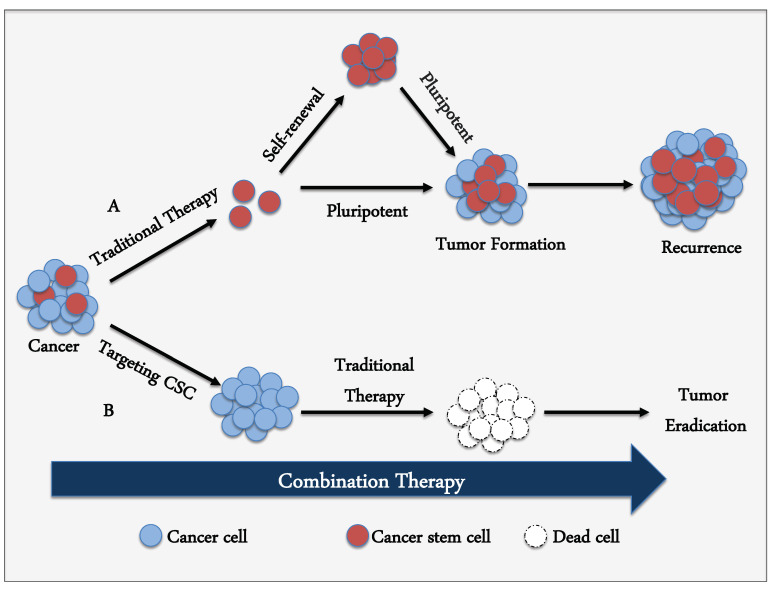
Combination therapy between traditional cytotoxic drugs with cancer stem cell (CSC)-targeting agents. (Path **A**) Chemotherapeutic and molecular-targeted drugs can attack most cancer cells, but CSCs can avoid these agents, leading to tumor regrowth. (Path **B**) Combination therapy between traditional drugs and CSC-targeting agents is predicted to be more effective.

The heterogeneity and high plasticity in cancer due to the presence of non-CSC tumor cells and CSCs have hindered efforts to detect appropriate targets for diverse cancers and establish a CSC-targeting strategy [40]. Therefore, the combination of traditional anticancer drugs with CSC-targeting agents has been evaluated (Figure 1) [6,39]. Nevertheless, plasticity still helps non-CSCs transdifferentiate into CSCs, hindering CSC-specific targeting. The identification of novel CSC-specific molecules and/or pathways taking plasticity into consideration is necessary [63]. The characteristics of CSCs should be investigated in more detail for the advancement of effective therapies targeting CSCs.

## 5. Pharmacology and the Mechanism of Action of Paclitaxel

**Figure 2:**
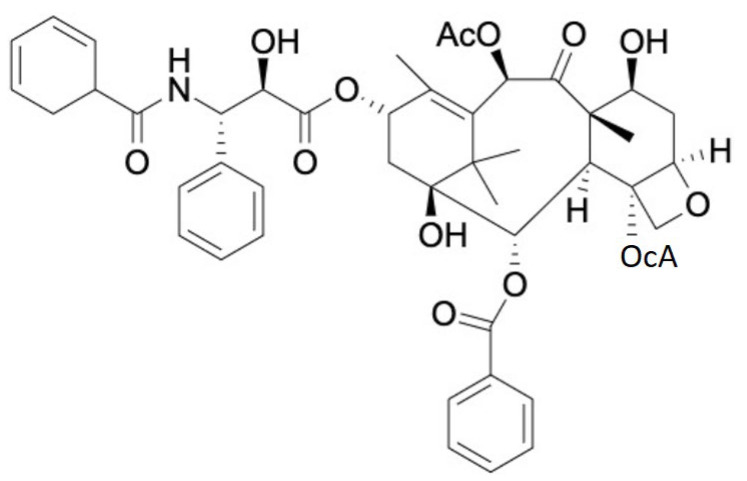
The structure of PTX.

The profound and unique properties of PTX have been previously studied [36]. PTX has been used in the treatment of various cancers such as breast cancer, colorectal cancer, squamous cell carcinoma in the urinary bladder, head and neck cancers, non-small-cell lung cancers (NSCLCs), and AIDS [65]. PTX is used to treat not only cancers but also other diseases, such as coronary heart disease, skin disorders, renal and hepatic fibrosis, inflammation, and axon regeneration, including degenerative brain diseases [66]. PTX is a member of the taxane family of anticancer drugs, along with docetaxel [67]. PTX is a tricyclic diterpenoid compound with a molecular formula of C47H51NO14, the chemical structure of which is shown in Figure 2. Due to its extraordinary hydrophobic character, PTX is most likely integrated into the hydrophobic space of the lipid bilayers of the cellular membrane, penetrating into the cytoplasm [18,68].

PTX stabilizes the assembly of tubulin into microtubules and prevents the dynamism of microtubules prohibiting cell cycle progression and blocking mitosis [69,70,71]. Once it enters into the cytoplasm, PTX binds to beta-tubulin and stabilizes microtubules by promoting the assembly of alpha- and beta-tubulin subunits, which are the building blocks of microtubules [69,72,73,74]. The drug reduces the critical dynamics of tubulin required for cell division [20]. Cell division halted at the G2 or M phase induces apoptosis due to the mitotic checkpoint. Calcium and low temperatures will maintain the dynamics of the microtubules, reducing the affinity of PTX for tubulin [36,69,75].

PTX cytotoxicity in vitro depends on its concentration [68,76,77]. The proliferation of human lung carcinoma cell line A549 cells, as well as human breast cancer cell line MCF-7 cells, was arrested at G2/M via the treatment with PTX at concentrations of approximately 3 to 12 nM, resulting in programmed cell death. A low dose of PTX was independently assessed for cancer cell invasiveness [78]. In this in vitro study, while 10 nM PTX was a non-anti-mitotic concentration in human breast cancer cell line MDA-MB-231 cells, the trans-well invasion of the cells was reduced at this dose by regulating the expression of voltage-dependent sodium channels. Additionally, low doses of PTX at 20 nM upregulated the expression of E-cadherin and downregulated that of β-catenin, leading to the suppression of tumor growth, metastasis, and angiogenesis in breast cancer when combined with a Wnt signaling inhibitor XAV939 [79].

**Figure 3:**
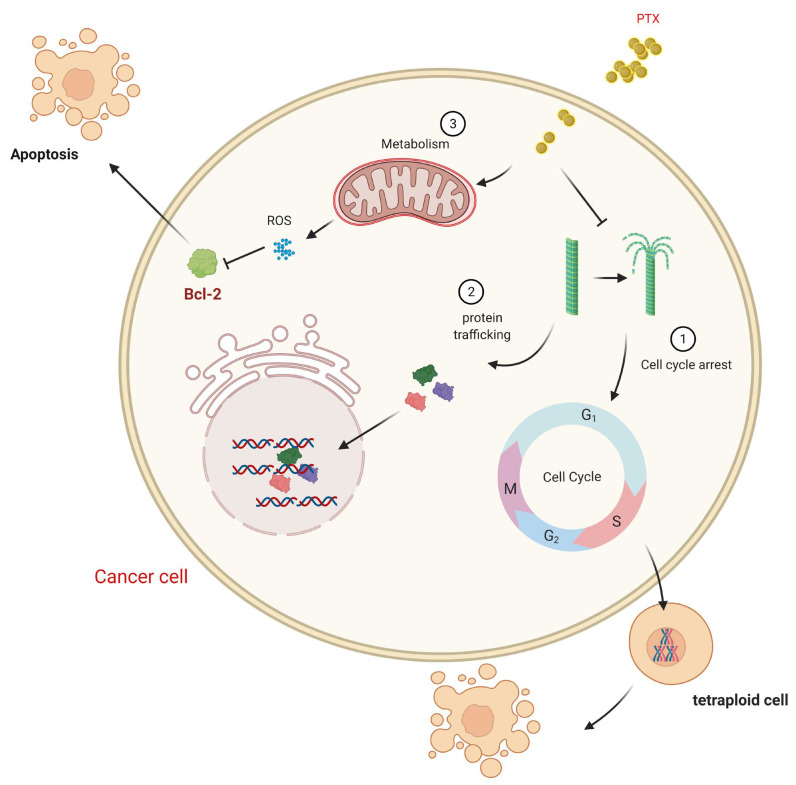
The mechanisms of PTX cytotoxicity in the cell. PTX can act through different mechanisms. After entering the cell, PTX can act as an antimicrotubular agent, leading to two actions: (1) cell cycle arrest, producing tetraploid cells containing 4×, in which unstable tetraploid cells undergo cell death; (2) disbalanced microtubule formation affects the protein traffic into the nucleus, especially the transcription factors necessary for cell survival or proliferation. PTX can also affect metabolism in mitochondria, elevating ROS levels, which inhibits Bcl-2 inducing apoptosis.

While PTX mainly induces the apoptosis targeting tubulin, PTX has been found to target mitochondria and inhibit the function of the apoptotic inhibitor protein B-cell Leukemia 2 (Bcl-2) [67]. However, there is a controversial situation arising regarding the phosphorylation of Bcl-2 affected by PTX. Some researchers have reported that the cytotoxicity of PTX retained its ability to cause Bcl-2 hyperphosphorylation, while others reported that the dephosphorylation of Bcl-2 coincided with apoptosis (Figure 3) [80,81,82]. Apoptosis does not immediately occur after exposure to PTX, while the duration of the exposure and constant Bcl-2 phosphorylation appeared to contribute to the drug’s cytotoxicity [83]. On the other hand, phosphorylated Bcl-2 was independently proven not to dimerize with BAX. In this context, the unassociated BAX is responsible for apoptosis on the phosphorylation of Bcl-2 [81]. Furthermore, there is an indication that PTX may block the cell cycle at G1 (Figure 3), activating the mitogen-activated protein/microtubule-associated protein (MAP) kinase, increasing the production of tumor necrosis factor and activating liposaccharide-inducible genes [84,85]. The transcriptional upregulation of the interleukin-1 upon treatment with PTX also has been reported, and is probably associated with its ability to activate nuclear factor κB (NF-κB) [86,87]. PTX induced the expression of the tumor suppressor protein p53 and the cyclin-dependent kinase inhibitor in the presence of a functional c-raf-1 (an upstream regulator of MAP kinase) [88].

## 6. Paclitaxel in Drug Resistance

**Figure 4:**
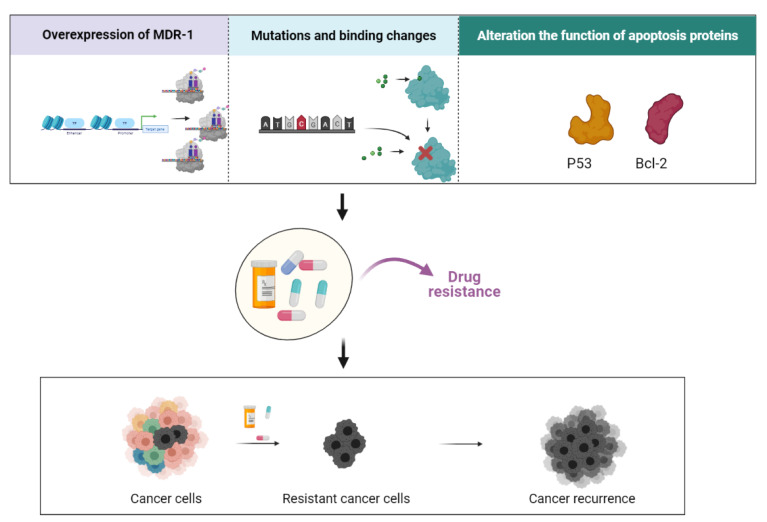
The representative figure shows different mechanisms of drug resistance caused by PTX. Drug resistance can occur due to the overexpression of multidrug resistance mutation 1 (MDR-1), mutations in the PTX binding site, or alterations in the functions or expression of different proteins that facilitate apoptosis. The drug-resistant cells are selected via drug treatment, and lead to cancer recurrence.

The development of drug resistance is one of the major limitations in anticancer therapies. Specifically, PTX has been found to enhance multidrug resistance (MDR) through three different procedures (Figure 4). The first is the overexpression of the MDR-1 gene, which is considered to be one of the adenosine triphosphate (ATP)-binding cassette (ABC) genes. MDR-1 encodes P-glycoprotein (P-gp), which is known as the drug transporting transmembrane ATP-dependent drug efflux pump. The MDR-1 gene is expressed in a wide variety of tumors and normal tissues [89,90,91,92,93,94].

PTX is reported to be a substrate of P-gp, of which overexpression results in the induction of PTX resistance. Because PTX accumulated in the brain and gut of P-gp knockout mice, it was determined that P-gp prevents PTX from passing through the blood–brain barrier and stops biliary elimination from the gut [90,95]. The quantitative polymerase chain reaction (PCR) proved a correlation between MDR-1 expression and the sensitivity to several drugs in the National Cancer Institute (NCI) anticancer drug screening panel. Sensitivity to PTX had a high negative correlation coefficient (−0.896) with MDR-1 expression [96].

Many studies have shown that there are increased levels of either MDR-1 mRNA [97,98,99] or P-gp itself in PTX-resistant cell lines [77,100,101]. However, the blocking of P-gp is partially sufficient to re-establish sensitivity to PTX, and this method was not remarkably effective in clinical trials [89,91].

The second is the alterations in the binding affinity of PTX to β-tubulin by the mutations in tubulin genes, which form a large multigene family encoding multiple tubulin isotypes with several posttranslational modifications. These mature proteins are considered to be the targets of anti-microtubule drugs [90]. The alterations in α and β tubulins, which have been described in PTX-resistant cell lines in vitro [102], are not yet known in every patient-derived tumor. There is still a possibility that PTX resistance is mediated in vivo by alterations in the levels of tubulin expression, or in the dynamics of tubulin polymerization adapted to the drug. Investigations on the differences in β-tubulin isotype expression and mutations in the β-tubulin genes in clinical samples to predict the response to PTX chemotherapy are technically very difficult and lead to different results [90]. An increase of the isotypes βI, βIII, and βIVa in epithelial tumors in the ovary was reportedly resistant to PTX via PCR with specific oligonucleotide primers [103]. Because the β-tubulin subtypes could alter microtubule dynamics in vitro [104], isotype composition may be a general mechanism of resistance to PTX [105]. The alterations in tubulins in several PTX-resistant cells are summarized in Table 1.

The last is the attenuation of apoptosis by proteins such as p53 and Bcl-2. Mutations in tumor suppressor gene (TSG) p53 are frequently found in human tumors, disturbing cells from growth arrest to induce apoptosis [111]. Mouse tumor cells conveying wild type p53 were significantly more sensitive to direct treatment with PTX than p53-deficient tumor cells [112]. In contrast, primary embryo fibroblasts with mutant p53 exhibited a significant increase in sensitivity to PTX [113]. Although the mechanism of sensitivity to the chemotherapeutic agents responsible for p53 genes is unknown, the inactivation of TSG p53 by DNA damage possibly appears to keep cells growing, resulting in drug resistance. In another study, Bcl-2 overexpression was shown to be oncogenic, increasing the resistance to drugs that induced apoptosis in some human cancer cells [114]. The overexpression of Bcl-2 is frequently found in prostate cancer and is considered to be associated with resistance to chemotherapy and hormonal therapy [115]. MAPs are also likely to be involved in the mechanism of resistance to drug-induced apoptosis. The expression of MAP4, which is negatively regulated by wild type p53, has been shown to increase sensitivity to PTX [116].

## 7. Combinatorial Therapy

Various studies have concentrated on the effect of PTX to enhance results among patients. Although PTX is one of the most effective and frequently used drugs for the treatment of different cancers, its efficiency is limited due to drug resistance. Therefore, PTX in combination with other therapeutic materials is considered. Radiation therapy is used in combinations in order to improve the therapeutic ratio for patients. Based on the early finding that radiation sensitivity occurs just before DNA replication begins, PTX was then theorized to be a potent radio-sensitizing agent due to its ability to arrest cells in the G2/M phase of the cell cycle, and it was further investigated for its ability to synergistically act with radiation [91,117,118]. The effect of drug–radiation therapy on human astrocytoma cell line G18 cells was successfully demonstrated by Tishler and colleagues, who reported that the sensitizer enhancement ratio was approximately 1.8, with 10% survival at 10 nM PTX [118]. Chemoradiotherapy with PTX improved the therapeutic outcome according to further studies both in vitro and in vivo [119,120]. However, Erlich and colleagues simultaneously revealed that gamma radiation during the G2/M phases showed radio-sensitization by PTX at 10 nM for the relatively radioresistant human cervical cancer cell line MS751, and C-33A was small when compared without PTX using radiation doses of a conventional fraction size [121,122]. The sensitizer enhancement ratio (SER) averaged 1.1 and 1.3 for the C-33A and MS751 cell lines, respectively [121].

Liebmann and colleges investigated the radio-sensitization properties of PTX in human breast cancer cell line MCF-7, lung carcinoma cell line A549, ovarian cancer cell line OVG-1, and pancreatic adenocarcinoma cell line PC-Sh using clonogenic assays and flow cytometry [123]. All of the cell lines were arrested at the G2/M phase after exposure to PTX ranging from 0 to 10 µM. However, the degree of radiosensitization by PTX varied depending on the human cancer cell line. The SER of PTX at 10% survival was 1.8, 1.6, and 1.5 in the MCF-7, OVG-1, and PC-Sh cells, respectively, while the pancreatic non-cancer cells did not show a radiosensitization response to PTX. On the other hand, PTX was unable to enhance the radiation sensitivity of the A549 cells at any concentration, even when combined with a protein synthesis inhibitor, cycloheximide. Considering that A549 cells are resistant to PTX, radio sensitization may not always improve the result via PTX combination. Including this point, the results reported by Erlich and colleagues could be revisited with respect to radio sensitization.

Many trials have been carried out to determine the combinatorial benefits of PTX with Thymoquinone (TQ), which shows significant evidence of anticancer effects. The treatment with the TQ–PTX combination differentially induced the expression of genes involved in the apoptosis cascade, p53 signaling, and JAK-STAT signaling in a triple-negative breast cancer cell line [124]. In an independent study, the anticancer effect of PTX against MCF-7 cells was shown to be effective when PTX was encapsulated together with TQ in nanoparticles in breast cancer treatment [125]. Very recently, the TQ–PTX combination was investigated against MCF-7 and T47D breast cancer cell lines [126]. This combination significantly increased the apoptotic/necrotic cell death percentages in T47D, and significantly induced autophagy in MCF-7 cells. In these cases, TQ appears to maintain PTX at a low dose, reducing the side-effect, but the mutual effect has not been investigated to date.

The combination treatment of the cells with salinomycin (SM) and PTX resulted in the marked cleavage of PARP and the induction of apoptosis, which was not observed following the treatment of the cells with either drug alone [127]. Nanoparticles encapsulating SM prevented metastasis in an orthotopic transplant model of breast cancer and, more significantly, improved the survival of mice when combined with PTX nanoparticles [128]. HA-decorated nanoparticles encapsulating SM and PTX successfully amplified the effect of chemotherapy, blocking CD44-positive CSCs [129,130].

Treatment with a combination of dasatinib and PTX not only decreased the proportion of breast CSCs in the tumor tissue, suppressing their self-renewal capacity, but also synergistically reduced the cell viability of PTX-resistant cells [131]. In vivo studies further demonstrated the effectiveness of the dasatinib-PTX combination in the inhibition of breast tumor growth.

In combination with a weekly dose of PTX 80 mg/m^2^, the maximum tolerant dose of dasatinib was raised to 120 mg/m^2^ [132]. The side-effects of the combination are consistent with prior experience of the monotherapy with each agent. Preliminary evidence of the antitumor effect of this combination was observed in patients with metastatic breast cancer, including patients with prior exposure to taxane. All of these data suggest that dasatinib is a promising agent for anti-breast cancer stem cells, and that it may overcome the resistance to chemotherapy in triple-negative breast cancer when combined with PTX.

Dasatinib combined with PTX also enhanced the inhibition of the colony formation of pancreatic cancer cells when compared with single-agent monotherapy. This combination effectively inhibited the phosphorylation of SRC, STAT3, AKT, and/or ERK in these pancreatic cancer cells. Therefore, the combination of dasatinib and PTX may be conceivable as a therapeutic approach for human pancreatic cancer. [133].

The combination of sorafenib (Sor) and PTX was demonstrated to have a positive effect on anti-angiogenesis in vivo in metastatic breast cancer [134]. A triple combination of radiation, Sor, and PTX has been reported to be effective on breast cancer cell lines [135]. Further investigation for the effects of radiation and the combination of Sor and PTX on CSCs will be interesting as CSCs are resistant to radiation therapy. Nawara et al. showed that the Sor-PTX combination enhanced the efficiency when compared to the monotherapy, demonstrating the combinatorial effects on CSCs [52,136] found in a synergistic or an additive manner. In this study, the Sor–PTX combination in low concentrations was evaluated to target CSCs, and significant suppression of the CSCs’ properties was found. These results pose a novel approach for targeting CSCs with anticancer drugs in low doses, which could effectively reduce the toxic side effects of chemotherapy [137].

Silibinin (SBN) has conventionally been applied for the treatment of liver diseases. The combination of SBN and PTX has been shown to be more efficient than the exclusive application of PTX or SBN in the treatment of human ovarian cancer cell line SKOV-3 cells [138]. Their results also strongly suggest that SBN inhibits the proliferation of SKOV-3 cells, and that the combination of SBN-PTX is more effective than PTX alone. The expression of p53 and p21 as apoptosis genes were simultaneously studied, showing that the genes were upregulated in the cells treated with the SBN–PTX combination when compared to those in the non-treated group.

The SBN–PTX combination also exhibited major anti-neoplastic effects in a diversity of cancer models such as skin, breast, colon, prostate, and kidney carcinomas [139]. SBN was available to reduce the side effects of PTX because SBN was a nontoxic antitumor agent, while PTX induced apoptosis [140,141,142,143,144,145].

Liu et al. in 2016 studied the effects of curcumin and PTX. The combined treatment of human oral squamous carcinoma cell line CAL 27 cells significantly inhibited cell growth and induced apoptosis via the decrease of the expression of Bcl-2 coupled with the increase of the expression of Bax, resulting in the increase of the relative ratio of Bcl-2/Bax to activate caspase-3. Thus, curcumin and PTX significantly inhibited cell growth and mediated cell apoptosis when compared to the results obtained from either single treatment [146].

Many studies have indicated that the combination of PTX and curcumin was quite effective for the treatment of cervical cancer in preclinical trials [147,148,149,150,151]. Curcumin has been demonstrated to sensitize PTX-induced apoptosis, enhancing the expression of p53 to activate caspase −3, −7, −8, and −9, to cleave poly (ADP-ribose) polymerase (PARP), and to release cytochrome c via Western blot analysis [147,149]. The combination of PTX and curcumin blocked cell cycle arrest at G2/M in human bladder cancer cells [152], and also synergistically enhanced PTX-induced apoptosis in lung cancer H1299 cells, inhibiting cell growth at low IC50 values via the combination when compared to PTX or curcumin alone [153].

Although PTX is approved and widely used for the clinical treatment of breast and ovarian cancers, various studies have reported that the combination of PTX and curcumin is more effective on breast, ovarian, brain, prostate and liver cancers than the exclusive use of PTX or curcumin, suggesting a synergistic effect [152,153,154,155,156,157,158,159,160,161,162,163,164,165,166,167,168].

The combination of PTX and curcumin, as well as either agent alone, was found to reduce lung metastasis in vivo [169]. On the other hand, PTX induced the expression of NF-kB in vitro, whereas curcumin suppressed it. With a relatively less-effective dose of PTX, the addition of curcumin resulted in effective antimetastatic therapy.

In another combination treatment, PTX and quercetin (Que) were used to treat prostate cancer. This combination exhibited the drastic inhibition of cell proliferation, cell cycle arrest at the G2/M phase, the inhibition of cell migration, and increased apoptosis and ROS generation. Quercetin increased the cancer-cell-killing effects of PTX, with nearly no side effects compared to the monotherapy of the PTX group [170]. This combination is expected to exert the most useful therapeutic effects.

Min et al. (2018) demonstrated the efficacy of the combination of caffeic acid (CA) with PTX as a cooperative anticancer action, the effect of which is probably determined by the MAPK signaling pathway and caspases. As a result, this synergistic effect was recognized as a strong inhibitory effect against the growth of non-small-cell lung cancer cell (NSCLC) line H1299 cells in vitro and in vivo. After CA treatment, cells were arrested in the intermediate phase between G1 and S phase, increasing the activities of both caspase-3 and caspase-9, leading to the enhancement of apoptosis. Furthermore, CA increased in vitro the events induced by PTX, such as the activation of Bax, Bid, and the downstream of Poly (ADP-ribose) polymerase-1 (PARP-1) cleavage, and the phosphorylation of extracellularly regulated kinase (Erk) 1/2 and c-Jun N-terminal protein kinase (JNK) 1/2. The combined treatment with CA and PTX exerted a more effective suppressive effect in vivo on the tumor growth of H1299 cell xenografts without significant adverse effects. Taken together, PTX treatment with a low dose of CA would result in a strong suppressive effect on the growth of NSCLC [171].

The effects of PTX in combination with withaferin A (WFA) on the growth, proliferation, migration, and invasion of human NSCLC cells have also been proposed by Kyakulaga and his colleagues. PTX and WFA synergistically inhibited colony formation, migration, and invasion whilst also increasing the induction of apoptosis in H1299 and A549 cells. Importantly, PTX was effective with WFA on PTX-resistant A549 cells, as well as PTX-sensitive A549 cells both in vitro and in vivo. Thus, the sensitivity of H1299 and A549 cells to the treatment was shown to be greater in the combination of PTX with WFA than in the single use of either PTX or WFA alone. These findings validate the use of WFA alone or with PTX in NSCLC cells and justify the further testing of clinically relevant models with the combination of PTX and WFA of advanced NSCLC as an alternative to current therapeutic strategies [172].

A successful translation of an alternate dosing strategy combining palbociclib and PTX in patients with advanced breast cancer was shown to be feasible and safe without evidence of additive toxicity. However, this combination requires further study in a larger randomized clinical trial with a direct comparison to single-agent PTX to determine whether or not this strategy finally improves outcomes for patients with advanced breast cancer [173].

The combination of PTX and doxorubicin (DOX) has been widely clinically used despite its serious toxicity. Yu and colleagues have successfully synthesized a prodrug PTX-S-DOX (PSD), the cytotoxicity of which has been demonstrated in vitro in comparison to the mixture of free PTX and DOX. PSD is favorable to enhance the anti-cancer effect and decrease harmful effects, improving the pharmacokinetics and anti-tumor properties. Because copper ions (Cu2+) could be organized in the anthracene nucleus of DOX, the prodrug PSD is hypothesized to be loaded into liposomes by the Cu2+ gradient. Therefore, combination chemotherapy was designed with the liposomes encapsulating PSD (PSD LPs) for controlled release. The PSD LPs enhance the accumulation of PSD in the tumor, showing more anti-tumor effects than that of the non-liposomal formulation of PSD [174].

Many clinical trials are still investigating the potential effects of paclitaxel for the treatment of different stages of cancers, as a monotherapy or in combination with a wide range of other treatments. In a clinical trials database, by 2021, there were more than 3700 registered clinical trials using paclitaxel for cancer. Among these trials, 43 reached or passed phase 4, while 700 were in phase 3. Some of these clinical trials are presented in Table 2. Collectively, various combinations with PTX could exert synergistic anticancer effects, demonstrating a promising regimen for the treatment of different types of cancer.

Collectively, various combinations with PTX could exert a synergistic anticancer effect and could be a promising regimen for the treatment of different types of cancer.

## 8. Conclusions

Combinations of different anticancer agents will be one solution for improving the efficacy of conventional chemotherapy, reducing side effects and avoiding MDR. Given the hierarchical complexity due to cancer stem cells, a strategy involving combination therapy could be useful to simultaneously target both the bulk of differentiated cancer cells and the minor population of cancer stem cells. In this context, the presence of cancer stem cells should be taken into consideration in order to evaluate the drug effects in search of more efficient drugs. Combination therapy may yield novel chemotherapy strategies in the future.

## Figures and Tables

**Table 1:** PTX-resistant cell lines with modifications and alternations in tubulins.

Cell Line	Cancer/Tissue Type	Affected Tubulin	Modification	PTX Resistance	Reference
**1A9PTX10 &1A9PTX22**	Ovarian cancer	βI	mutation	accelerating	[106]
**A549**	Non-small-cell lung cancer	βIVa, βIII, βI	Altered expression	accelerating	[103]
**H69/Txl**	Small-cell lung cancer	α-tubulin	acetylation	accelerating	[107]
**Pac 10**	Prostate carcinoma	βIII, βIVa	Altered expression	accelerating	[108]
**KPTA5**	Leukemia	βIVa	Altered expression	accelerating	[109]
**MES-SA**	Sarcoma	βIII, βIVa	Altered expression	reducing	[110]

**Table 2:** Clinical trials involving PTX.

Combination	Phase	Cancer Type	Clinical Trial Identifier
Napabucasin and Gemcitabine	3	Metastatic pancreatic cancer	NCT03721744
Bevacizumab	3	Metastatic breast cancer	NCT00028990
Fruquintinib	3	Gastric cancer	NCT03223376
NovoTTF-100L	3	Ovarian cancer	NCT03940196
Atezolizumab	3	Triple negative breast cancer	NCT02425891
Cisplatin plus radiotherapy	4	Non-small-cell lung cancer	NCT00686322
Chemotherapy (Carboplatin)	4	Her-2 negative breast cancer	NCT03799692
RAD001 and Carboplatin	4	Carcinoma, large cell Neuroendocrine tumors	NCT01317615
Bevacizumab and Carboplatin	4	Ovarian cancer	NCT01706120
Bevacizumab	4	Triple negative breast cancer	NCT01094184
Vantictumab	1	Metastatic breast cancer	NCT01973309
Cisplatin	2	Esophageal cancer	NCT01444547
Lapatinib	2	Urothelial cancer and bladder cancer	NCT01700010
Reparixin	2	Metastatic breast cancer	NCT02370238
Tegafur, Oxaliplatin and Capecitabine	3	Stomach cancer	NCT04135781
DHP107	2	Recurrent or metastatic breast cancer	NCT03326102
Lenalidomide	1	Prostate cancer	NCT00933426
LDE225	1	Recurrent ovarian cancer	NCT02195973
Cirmtuzumab	1	Breast neoplasms	NCT02776917

## Data Availability

Not applicable.
